# In Vivo and Impression Cytology Study on the Effect of Compatible Solutes Eye Drops on the Ocular Surface Epithelial Cell Quality in Dry Eye Patients

**DOI:** 10.1155/2015/351424

**Published:** 2015-06-29

**Authors:** Manuela Lanzini, Claudia Curcio, Rossella Annamaria Colabelli-Gisoldi, Alessandra Mastropasqua, Roberta Calienno, Luca Agnifili, Mario Nubile, Leonardo Mastropasqua

**Affiliations:** ^1^Department of Medicine and Ageing Sciences, Ophthalmology Clinic, University “G. d'Annunzio” of Chieti-Pescara, 66100 Chieti, Italy; ^2^Department of Medicine and Ageing Science, Biological Eye Center, CeSI, University “G. d'Annunzio” of Chieti-Pescara, 66100 Chieti, Italy; ^3^Azienda Ospedaliera San Giovanni-Addolorata, 00184 Roma, Italy; ^4^Ophthalmology Clinic, Campus Biomedico, 00185 Rome, Italy

## Abstract

The aim of this study is to investigate in vivo and ex vivo ocular surface alterations induced by dry eye disease and modification after osmoprotective therapy. Forty-eight eyes of 24 patients suffering from dry eye have been recruited. All patients received Optive (compatible solutes) eye drops in one randomly selected eye and Hylogel (sodium hyaluronate 0,2%) in the other. Follow-up included a baseline visit and further examination 30-, 60-, and 90-day intervals (which comprises clinical evaluation, in vivo confocal microscopy—IVCM—of the ocular surface, and conjunctival impression cytology). No significant difference in Schirmer I Test, TBUT, and vital staining results was observed during the follow-up period in both groups. IVCM showed in all patients an improvement of ocular surface epithelial morphology and signs of inflammation (oedema and keratocyte activation). However, these modifications were more evident in patients treated with Optive therapy. A significant reduction of the expression of MMP9 and IL6 in Optive group was observed during the follow-up period in comparison to Hylogel treatment. Our results show that in dry eye disease therapy based on osmoprotective eye drops determines a reduction of inflammatory activation of ocular surface, with consequent improvement of the quality of corneal and conjunctival epithelium.

## 1. Introduction

Dry eye syndrome (DES) is a multifactorial disease of the functional lacrimal unit, formed by lacrimal gland, adnexae, tear film, and ocular surface. Dry eye alters and damages corneal and conjunctival epithelium, producing specific symptoms and visual disturbances [1].

In dry eye patients, the ocular surface becomes diffusely irregular with consequent differentiation of the epithelial cells. DES can produce an irregularity of corneal epithelial continuity with punctate or widespread defects. In conjunctiva there is also a reduction of goblet cell density [2].

The exact mechanism by which these changes occur has not been clarified, but it may be linked to hyperosmolar tear film stress. The osmotic stress causes a modification of corneal epithelium which determines an increase of the stratum corneum, precursors of cellular coating [2].

Chronic inflammation and hyperosmolarity play an important role in the genesis and maintenance of dry eye, since they are both the cause and the consequence in a pathological self-renewing cycle [1].

The osmolarity of the tear film is determined by the presence of cations (sodium, potassium, calcium, magnesium, and iron) and anions (chloride, bicarbonate, and phosphate) [3].

It has been shown that the cellular hypertonicity of different organisms could be compensated by compatible solutes, which balance the osmotic pressure and do not interfere with cellular metabolism [4]. These solutes include amino acids, carbohydrates, methylamine, and urea [4, 5]. They are also considered osmoprotective substances which can improve cellular survival under hypertonicity conditions. These molecules are used by cells to introduce liquids in the intracellular area and maintain cellular volume [5].

Experimental data, obtained on cultured corneal epithelial cells, show that compatible solutes, as L-carnitine, erythritol, and betaine, can suppress matrix metalloproteinase (MMP) production and activation [6] and expression of proinflammatory cytokines (TNF-*α*, IL-1*β*, and IL-6) and chemokines (IL-8, CCL2, and CCL20) [7] without any modifications of cellular metabolism. In fact, they are able to hydrate and induce an osmoprotective effect on the ocular surface. These macromolecules introduce liquids in the intracellular area producing a direct hydration and a natural osmoprotection [6, 8].

The aim of this study is to investigate, in vivo and ex vivo, ocular surface alterations and inflammation in DES and modifications related to the use of compatible solutes eye drops.

## 2. Material and Methods

### 2.1. Patient's Enrolment and Study Design

This study was conducted at the Department of Medicine and Ageing Science of the G. d'Annunzio University of Chieti-Pescara, Italy. The protocol used was approved by the institutional review and the study was conducted in concordance with the tenets of the Declaration of Helsinki. Informed consent was obtained from all the participants after explanation of the nature and the possible consequences of the study.

48 eyes of 24 patients, 15 women and 9 men, age range 19–64 years, affected by DES have been recruited.

Inclusion criteria were as follows: current use of artificial tears, Schirmer I Test < 8 mm, and tear break-up time (TBUT) < 8 seconds. Exclusion criteria were as follows: age <18 years, pregnant or lactating women, ocular surgery within the last three months, presence of eye infection, presence of glaucoma, and inadequate lid closure.

Recruited patients were treated by using a combination of sodium carboxymethylcellulose (0.5%) and glycerol (0.9%) eye drops (Optive, Allergan Inc.) 4 times daily for 90 days in one randomly selected eye, and the comparator eye (control eye) underwent therapy with sodium hyaluronate 0,2% eye drops (Hylogel, Visufarma spa) 4 times daily for 90 days.

Patients were followed up at 30, 60, and 90 days.

At the baseline and follow-up visits, the following parameters have been assessed: Schirmer I Test, TBUT, lissamine green staining of the ocular surface graded by the Lemp classification [9], in vivo confocal microscopy (IVCM), and impression cytology of bulbar conjunctiva.

### 2.2. In Vivo Confocal Microscopy

At each follow-up visit, microscopic assessment of the ocular surface epithelial health was performed by using IVCM (Rostok Cornea Module-HRT 2, Heidelberg).

In each eye examined, at least 100 images of corneal epithelium and 100 images of conjunctival bulbar epithelium in nasal and temporal sector were acquired.

The analysed parameters were regularity of the corneal and conjunctival epithelium and evaluation of inflammation within the ocular surface. The presence of inflammatory cells in subepithelial layer and anterior stroma was considered as a marker of inflammatory activation. Adobe Photoshop program (Adobe Systems Inc., Jose, CA) was used to define picks of reflectivity from confocal microscopy images. Stromal reflectivity was considered an indirect sign of the presence of oedema and keratocyte activation.

### 2.3. Impression Cytology

Conjunctival impression cytology samples were collected at each follow-up visit using Millicell-CM 0.4*μ*m (Millipore, Bedford, MA) and the cells were fixed with cytology fixative (Biofix, Bio Optica, Milano, Italy).

The Millicell membranes were hydrated with distilled water; 80% alcohol was added for 2 min. The membranes were washed in distilled water and Phosphate Buffered Saline (PBS) was added for 2 min, followed by 2 washes with Wash Buffer (Dako, Glostrup, Denmark) of 2 min each. Then, Ribonuclease A (Sigma-Aldrich, St. Louis, MO) diluted 1 : 290 in PBS was incubated for 20 min at room temperature. The specimens were washed and PBS-BSA 1% was added for 1 hour at room temperature. Finally, MMP9 antibody (Dako) diluted 1 : 50 and IL6 antibody (Abcam, Cambridge, UK) at 1 : 200 both in antibody diluent (Dako) were incubated overnight at 4°C. Samples were washed and anti-rabbit Alexa fluor 488 (Invitrogen, San Giuliano Milanese, Italy) diluted 1 : 200 and propidium iodide at 1 : 150 were added and incubated for 1 hour at room temperature. Membranes were mounted with a drop of Fluorescent Mounting Medium (Dako) and Zeiss Confocal LSM 510 (Carl Zeiss MicroImaging GmbH, Vertrieb, Germany) was used to visualize the cells.

Five different fields for each impression cytology sample were evaluated. Positive (red nucleus and green cytoplasm) and negative (red nucleus) cells were counted and the positivity percentage was calculated. All evaluations of impression cytology specimens were performed by two independent observers masked to the details of the staining technique used. Digital images of representative areas were taken.

### 2.4. Statistical Analysis

Differences in markers expression for selected antigens were assessed using the Student unpaired *t*-test (GraphPad Prism 5, GraphPad Software, San Diego, CA).

## 3. Results

### 3.1. Clinical Outcomes

Schirmer I Test and TBUT did not show a significant modification during the follow-up period, either in Optive or in Hylogel treated eyes; moreover, no significant differences were revealed between the two groups.

Lissamine green staining of the ocular surface evaluated by means of Lemp classification showed a significant improvement of ocular surface epithelial regularity in both groups at the end of follow-up period. No significant differences were observed between the two groups.

### 3.2. IVCM

**Figure 1:**
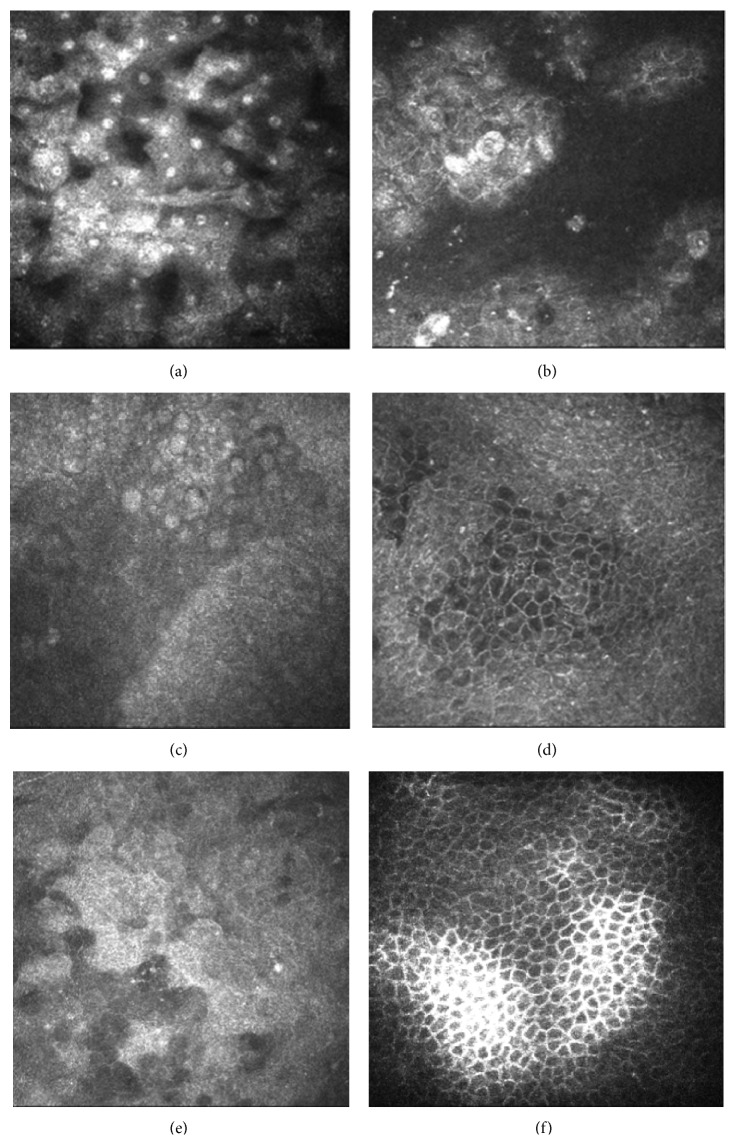
IVCM images of ocular surface in dry eye. (a) Squamous metaplasia of conjunctival epithelium. (b) Irregularity and desquamation of conjunctival epithelium. (c–f) Improving of quality and regularity of conjunctival epithelial cells more evident after 90 days of Optive treatment (c-d) with respect to Hylogel group (e-f).

In both groups, the morphology of corneal and conjunctival epithelia showed an improvement during the follow-up period. Comparing baseline images with findings at 30, 60, and 90 days after treatment, a decrease in reflectivity of superficial cells was observed together with an increase of regularity of cell shape and size. No morphological differences were revealed between the two groups (Figure 1).

**Figure 2:**
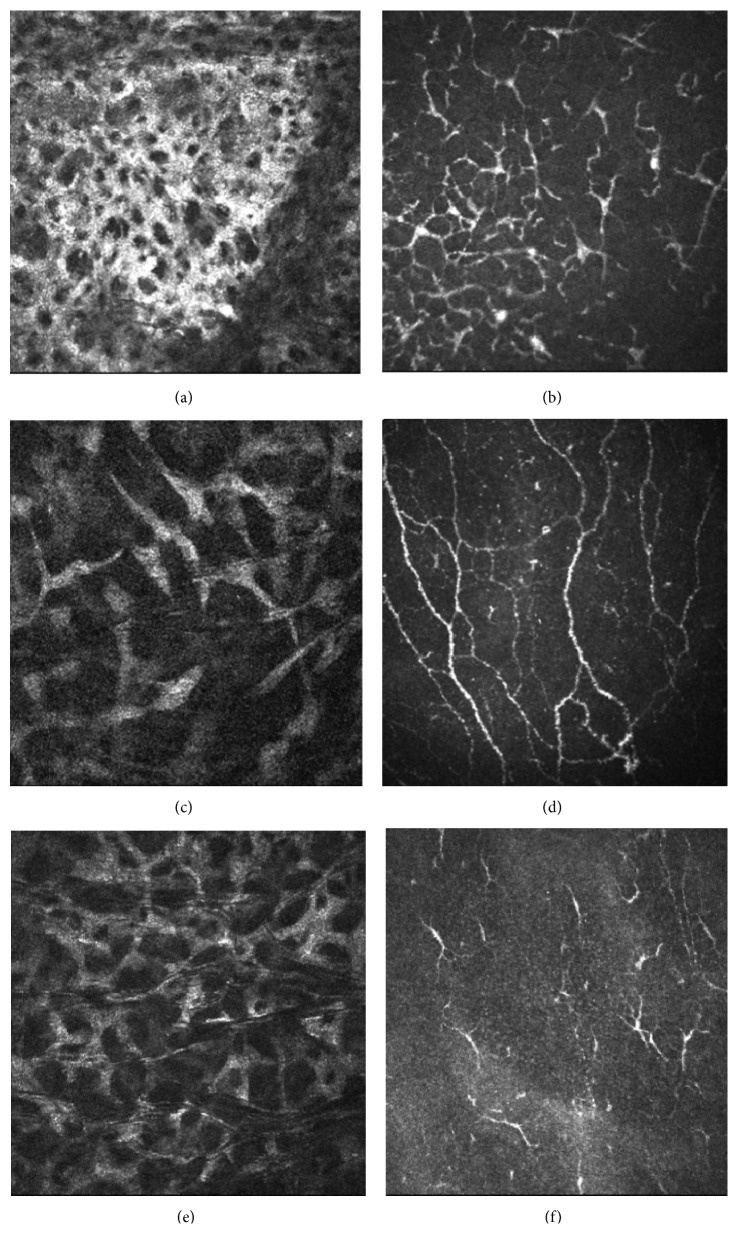
IVCM images of ocular surface inflammation in dry eye. (a) Numerous activated keratocytes in corneal anterior stroma. (b) High number of dendritic cells in corneal subepithelium. (c–f) Reduction of inflammation signs in corneal stroma and subepithelial layer, more evident after 90 days of Optive treatment (c-d) with respect to Hylogel group (e-f).

Stromal reflectivity showed a significant decrease both in Optive group (*p* = 0,0009) and in Hylogel group (*p* = 0,0400) at the end of follow-up period. Moreover, a significant difference was observed at 90 days comparing the two treatment groups (*p* = 0,0009) (Figure 2).

### 3.3. Impression Cytology: Evaluation of MMP9 and IL6 Expression

**Figure 3:**
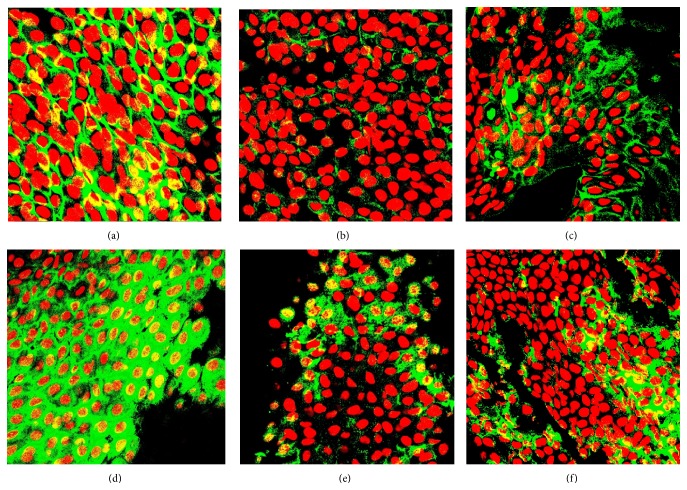
Immunofluorescence staining of impression cytology. (a–c) Cytoplasmic MMP9 staining in conjunctival cells at baseline (a) and its significant reduction observed after 90 days of Optive (b) and Hylogel (c) therapy. (d–f) Expression of IL6 in conjunctival cells at day 0 (d) and its downmodulation at the end of the treatment in Optive (e) and Hylogel (f) groups. Original magnification ×630.

The immunofluorescence staining of the conjunctival samples is shown in Figure 3.

**Figure 4:**
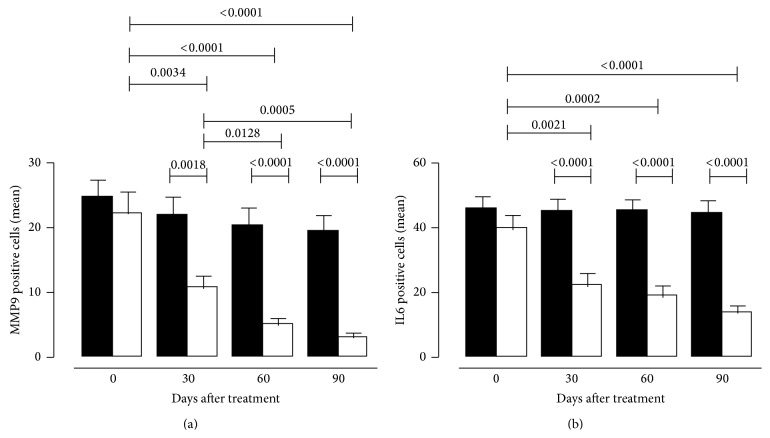
Statistical analysis of MMP9 (a) and IL6 (b) expression in Hylogel (black bars) and Optive (white bars) groups at baseline (day 0) and its variations during follow-up period (30, 60, and 90 days). Data are expressed as mean ± SEM. The unpaired Student's *t*-test was used to evaluate differences in markers expression. Significant *p* values are reported.

MMP9 expression was significantly downmodulated in Optive treated eyes at 30 (*p* = 0,0018) and 60 and 90 (*p* < 0,0001) days of therapy when comparing the two treatment groups (Figure 4(a)).

Comparing the variations of the expression of MMP9 from day 0 to day 90, a gradual but strong reduction was achieved in Optive group (*p* < 0,0001), while no significance was observed in Hylogel treated eyes (Figure 4(a)).

IL6 positivity in conjunctival samples revealed significant differences between Optive and Hylogel group when observed 30 (*p* < 0,0001), 60 (*p* < 0,0001), and 90 days (*p* < 0,0001) after treatment (Figure 4(b)). Moreover, a significant reduction was achieved among Optive treated eyes during all of the follow-up period (day 0 versus 90 *p* < 0,0001), while no modifications were observed in Hylogel group (Figure 4(b)).

## 4. Discussion

Hyperosmolarity represents an inflammatory stress to the limbal and corneal epithelium, inducing the expression of proinflammatory cytokines and metalloproteinase which produce an increase of desquamating cellular processes and nucleus/cytoplasm ratio and a reduction of cellular interconnections [10]. Tear film osmolarity depends on the dynamic modification of the tear film balance resulting from the production, retention, and elimination of tears. It was also related to the lacrimal flow and evaporation [2].

The increase of tear film osmolarity determines a reduction of conjunctival goblet cells, responsible for mucin production with consequent epithelial damage [11].

An in vivo study on mice, testing the effect of topical application of betaine, L-carnitine, and erythritol, showed a significant reduction in number of TUNEL-positive cells and expression of inflammatory mediators [12].

As already observed by Wei and colleagues, the inflammatory mediators associated with DES pathogenesis can be divided as follows: (i) ubiquitous inflammatory cytokines, (ii) Th1- and (iii) Th17-related cytokines, (iv) chemokines and their receptors, (v) metalloproteinase, and (vi) secretory phospholipases [13].

MMPs play a crucial role in initiating and maintaining ocular surface damage [14].

MMP9 is the most important gelatinase present on the ocular surface, and its levels seem to be higher in tears of patients with dry eye [15]. Desiccating stress was found to increase MMP9 in a murine model [16].

The conjunctival expression of MMP9 in DES patients is already assessed [17]. Moreover, MMPs are responsible for the extracellular matrix destruction observed in various diseases as arthritis, cancer, and autoimmune disorders [18, 19]. Under stress conditions, such as hyperosmolarity [11, 20], MMP9 is released by the ocular surface cells [21]. Furthermore, MMP9 has been demonstrated to accelerate corneal epithelial regeneration in the healing process by modulating the inflammatory response. Therefore, the increase of MMP9 activity on the ocular surface can amplify the chronic immune-based inflammation of dry eye [21].

In our study, the downmodulation of MMP9 expression in compatible solutes treated eye is linked to a clinical improvement of the ocular surface epithelia, suggesting a key role of MMP9 in the physiopathology of the disease.

The expression of IL6 was assessed as marker of conjunctival inflammation status. The increased IL6 levels in the ocular epithelium indicate an active immune state of the microenvironment [22].

In Sjögren syndrome, the ocular surface epithelium is a target of the autoimmune process [23], and increased levels of IL6 could be referred to as the direct involvement of the conjunctiva in the pathogenic mechanism of the disease, indicating a relevant role in ocular surface inflammatory damage [17].

Our data showed a decreased positivity for IL6 induced by osmoprotective treatment, suggesting an active downmodulation of inflammatory response.

The expression of MMP9 and IL6 in dry eye patients may indicate that inflammatory mechanisms play a synergistic role in the development of the disease.

The osmoprotectants compatible solutes, alone or in combination, were found to protect against stress activation of corneal epithelial cells cultured in hyperosmolar media [6–8].

Clinical studies on human subjects suffering from DES evidenced a noninferior efficacy and safety of the osmoprotective compatible solutes therapy with respect to standard sodium hyaluronate treatment [24].

In vivo confocal microscopy and immunofluorescence staining of impression cytology specimens were previously used in combination to assess modifications of conjunctival and corneal epithelia due to aging or limbal pathologies [25, 26].

Several discordant results are reported in literature about the real effect of hyaluronic acid compounds and Optive on BUT and Schirmer test values [24, 27–32]; however, difference in inclusion criteria, molecules concentration, and daily dosage may explain the variability of the outcome.

In our results, no changes were observed in BUT and Schirmer test values in both treatment groups, suggesting that both therapies are not able to increase the quantitative production of tears or to improve the lipidic component of the tear film. This finding may be related to our inclusion criteria of enrolled patients suffering from severe form of DES.

The real clinical effect of osmoprotection on ocular surface is therefore represented by an improvement of epithelia regularity as a consequence of better and specific inflammation control.

## 5. Conclusions

In conclusion, the ex vivo results of this study indicate that treatment based on compatible solutes eye drops in DES determines better control of ocular surface inflammatory activation, with respect to hyaluronic acid therapy. The effect of the decrease of inflammation is revealed in vivo by an improvement of corneal and conjunctival epithelium morphology together with a reduction of corneal stromal reflectivity. In view of this data, osmoprotective approach can represent, alone or in combination, a valid strategy in DES management.
